# First Record of the Scarab Beetle, *Phyllophaga lissopyge* from South America, with Descriptions of Adult Seasonal Activity and Male Response to Sex Attractants

**DOI:** 10.1673/031.011.0123

**Published:** 2011-02-25

**Authors:** Anuar Morales-Rodriguez, Daniel C. Peck, Paul S. Robbins

**Affiliations:** ^1^ Department of Plant Science and Plant Pathology, Montana State University, 10 Marsh Laboratory, Bozeman, MT 59717; ^2^ Department of Entomology, New York State Agricultural Experiment Station, Cornell University, 630 W. North Street, Geneva, NY 14456; ^3^ Present address: Subtropical Insects Research Unit, US Horticultural Research Laboratory, ARS, USDA, 2001 South Rock Rd., Ft. Pierce, FL 34949.

**Keywords:** Antioquia, methyl 2-(methylthio) benzoate, Coleoptera, Colombia, L-isoleucine, L-leucine, L-valine, Melolonthinae, methyl 2-(methylthio), pheromone, Scarabaeidae

## Abstract

Phyllophaga lissopyge (Bates) (Coleoptera: Scarabaeidae: Melolonthinae) is reported for the first time from South America. Male sex pheromone response is described for P. lissopyge and two other co-occurring Phyllophaga species. Adults of P. lissopyge and P. menetriesi (Blanchard) flew to traps baited with methyl 2-(methylthio) benzoate whereas adults of P. obsoleta (Blanchard) flew irregularly to four different pheromone compounds. Adult seasonal activity is described from males captures in Rionegro, Antioquia, Colombia.

## Resumen

Se reporta por primera vez Phyllophaga lissopyge (Bates) (Coleoptera: Scarabaeidae: Melolonthinae) en Sur América. Se describe la respuesta de los machos de P. lissopyge y de otras dos species de Phyllophaga recolectados en trampas cebadas con feromona sexual. Los adultos de P. lissopyge y P. menetriesi (Blanchard) volaron a las trampas cebadas con metil-2 - (metiltio) benzoate mientras los adultos de P. obsoleta (Blanchard) volaron irregularmente a cuatro diferentes compuestos de la feromona. Se describe la actividad estacional de los adultos basada en los machos capturados en trampas en Rionegro, Antioquia, Colombia.

## Introduction

The New World genus Phyllophaga (s. lato) (Coleoptera: Scarabaeidae: Melolonthinae) encompasses 865 extant species, including 442 from Central America, 217 from Canada and the United States, 186 from the Caribbean Islands, and 109 from South America (Evans and Smith 2009). Some overlap of species distribution occurs between these regions. Both larvae and adults of various Phyllophaga species are economically important pests of a variety of agricultural crops such as bean, cassava, coffee, corn, ornamentals, pasture grasses, peanut, pepper, potato, and sugarcane (King 1984, 1996a, 1996b; Morón 1986, 2006; Londoño 1993; Salvadori and Oliveira 2001; Salvadori and Silva 2004; Espinosa-Islas et al. 2005; Pardo-Locarno et al. 2005; Ortega-Ojeda et al. 2007).

Evans and Smith (2009) reported 28 species of Phyllophaga in Colombia (Table 1). Restrepo-Giraldo et al. (2003), however, reported 29 species in Colombia including P. sericata Blanchard, a species not reported by Evans and Smith (2009). Serna-Patiño (2004) added P. gigantea based on light-trap captures in Pereira, Risaralda. We report here the first capture of Phyllophaga lissopyge (Bates) (Coleoptera: Scarabidae) in South America, bringing the total number of species listed from Colombia to 31.

In Colombia, Phyllophaga flight activity generally occurs during the two rainy seasons, either March to May or September to November (Vallejo et al. 1998; Pardo-Locarno et al. 2002; Serna-Patiño 2004; Villegas et al. 2006, 2008). After emerging from the soil, females release sex pheromones by opening the pygidium and extruding a gland from which the pheromone volatilizes (for photos, see Leal et al. 1993; Nojima et al. 2003; Zarbin et al. 2007). Sex pheromones identified from the Phyllophaga include methyl 2-(methylthio) benzoate from P. crinita (Robbins et al. 2003); methyl esters of three amino acids, including L-valine, from P. anxia and P. (Phytalus) georgiana (Zhang et al. 1997; Robbins et al. 2009); L-isoleucine from P. anxia and P. elenans (Zhang et al. 1997; Leal et al. 2003); L-leucine from P. lanceolata, (Nojima et al. 2003); and phenol and p-cresol from P. cuyabana (Zarbin et al. 2007). Furthermore, an extensive trapping study by Robbins et al. (2006) demonstrated the widespread use of the methyl esters of L-valine and L-isoleucine methyl ester in the mate-finding systems of >50 species of Phyllophaga in the United States and Canada. This manuscript is the first part of a study of trapping Phyllophaga spp. with sex attractants in various regions of Colombia.

## Materials and Methods

The study site was located at the “La Selva” research station of the Corporación Colombiana de Investigación Agropecuaria in Rionegro, Antioquia, Colombia (latitude 6° 9′ 17.1792″, longitude -75° 22′ 52.845″, and 2150 m elevation). This site was chosen because of its history of abundant Phyllophaga populations. According to the Instituto de Hidrología, Meteorología y Estudios Ambientales station in Rionegro, Antioquia, the average temperature at the location is 17° C (8.0–25.0° C) and precipitation is 1800–2500 mm per year, largely falling in a bimodal pattern from April–May and September–October.

The methodology used in the present study was similar to prior studies (Zhang et al. 1997; Leal et al. 2003; Nojima et al. 2003; Robbins et al. 2003, 2006, 2008, 2009; Alm et al. 2004) that captured Phyllophaga adults using sex attractants. Cross-vane traps (see Robbins et al. 2006) were hung on a series of metal stakes such that the trap bottom was 1 m above the ground. A line of 12 traps, each separated by 20 m, were situated along the edge of a corn and cabbage field. Traps were maintained in the field from August 2003 to September 2004. Traps were emptied and rerandomized weekly. Pheromone lures were replaced every 4 weeks. Each trap was baited with an individual lure from the following: eight blends of the methyl esters of L-valine and L-isoleucine (100:0, 90:10, 80:20, 60:40, 40:60, 20:80, 10:90 and 0:100) (4 mg each rubber septa), L-leucine methyl ester (4 mg each rubber septa), methyl 2-amino benzoate (1 mg each rubber septa), methyl 2-(methylthio) benzoate (1 mg each rubber septa), and a control trap containing a blank septa. All lures were supplied by ChemTica Internacional, (www.chemtica.com). After collection, insects were frozen until identification. All specimens were identified to species using characteristics of the male genitalia (Morón 2001, 2003). The identification of P. lissopyge was confirmed by Dr. María Milagro Coca-Abia (Centro de Investigaciones y Tecnologías Agroalimentarias, Zaragoza, Spain) by comparison with museum specimens. Local rainfall data were obtained directly from the Instituto de Hidrología, Meteorología y Estudios Ambientales station in Rionegro, Antioquia.

**Table 1.**
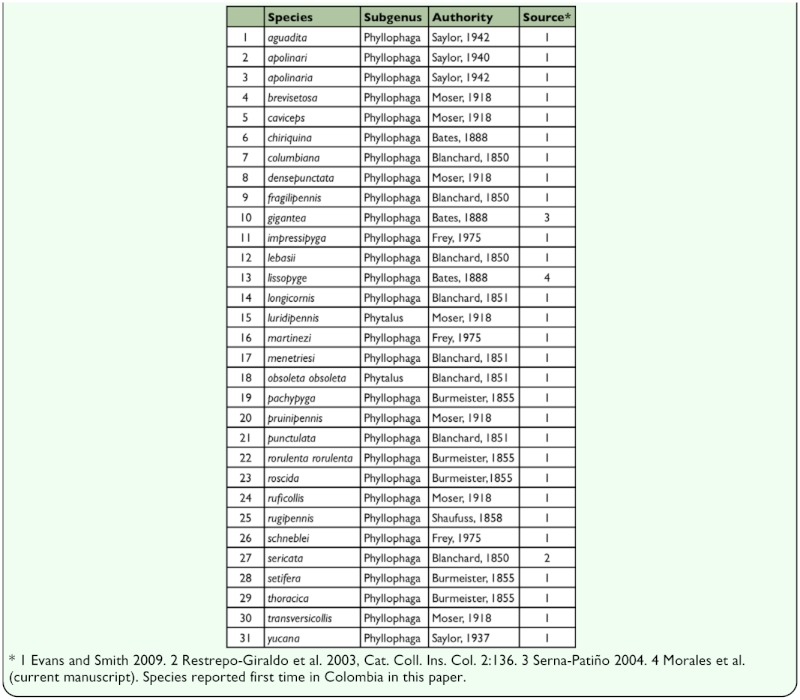
Checklist of the Phyllophaga from Colombia.

A male capture response curve was constructed for each Phyllophaga species to illustrate the proportional capture of males collected over the entire study with respect to pheromone treatment. The seasonal incidence of each species was also examined by plotting the number of captures versus weekly precipitation to reveal any correspondence between rainfall and adult activity.

## Results

### Material examined

A total of 156 males and no females were captured, representing three species of Phyllophaga. These included 135 P. (Phyllophaga) lissopyge, 20 P. (Phytalus) obsoleta, and 1 P. (Phyllophaga) menetriesi, representing 86.5, 12.8, and 0.7% of total captures, respectively. This is the first record of P. lissopyge from Colombia and South America.

**Figure 1.**
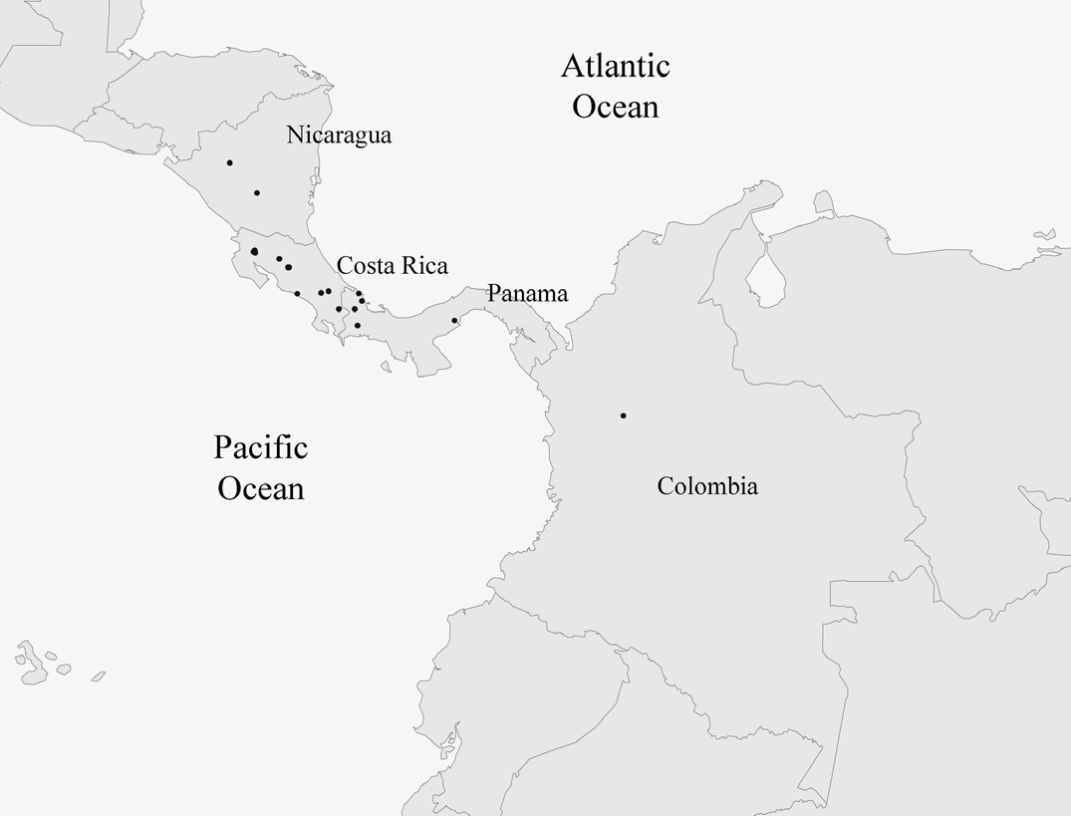
Geographic distribution of Phyllophaga lissopyge. High quality figures are available online.

### Male pheromone response

**Figure 2.**
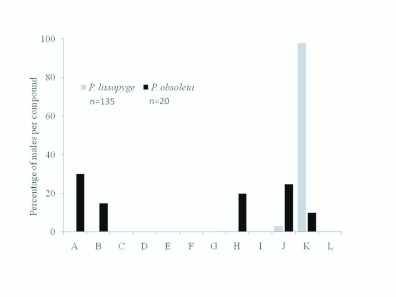
Proportional catch, by species, of adult Phyllophaga Hssopyge and P. obsoleta in traps with 12 different blends of pheromones in Rionegro, Antioquia, Colombia. (A) untreated check, (B) 100:0, (C) 90:10, (D) 80:20, (E) 60:40, (F) 40:60, (G) 20:80, (H) 10:90, and (I) 0:100 of L-valine methyl ester: L-isoleucine methyl ester; (J) L-leucine methyl ester, (K) methyl 2-(methylthio) benzoate, (L) methyl 2-amino benzoate. High quality figures are available online.

For P. lissopyge, male captures were greatest in those traps baited with methyl 2-(methylthio) benzoate (97.8%, n = 132); the remaining males (2.2%, n = 3) all flew to Lleucine methyl ester (Figure 2). In contrast, P. obsoleta showed no clear preference for any pheromone treatment; males were recovered from 100% L-valine methyl ester (15.0%, n = 3), 10:90 L-valine methyl ester: L-isoleucine methyl ester (20.0%, n = 4), L-leucine methyl ester (25.0%, n = 5), methyl 2-(methylthio) benzoate (10.0%, n = 2), and untreated check trap (30.0%, n = 6). The single male P. menetriesi was captured in a trap baited with methyl 2-(methylthio) benzoate.

### Seasonal incidence

**Figure 3.**
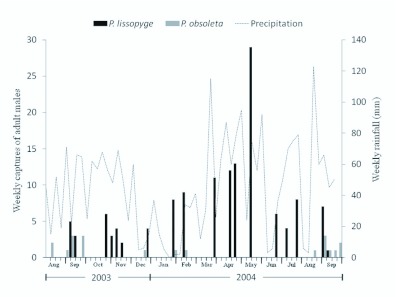
Numbers of adult Phyllophaga lissopyge and P. obsoleta collected weekly from pheromone traps with respect to weekly precipitation in Rionegro, Antioquia, Colombia. High quality figures are available online.

Adults of white grub species are commonly called “marceños” (March beetles) because many species fly in March. Phyllophaga lissopyge, however, was captured during every month of the year with the exception of August in both 2003 and 2004 (Figure 3). Almost half of all captures (48.1%) occurred during the four months of highest precipitation, March to June 2004 (Figure 3). Phyllophaga lissopyge adults were captured primarily after or during a period of high precipitation (Pearson's r = 0.64364; n = 14) with the exception of January 2004 (usually the driest month in the area), when 8 males were captured after the field was heavily irrigated.

Phyllophaga obsoleta males were captured mainly during August and September of both years (9 adults in 2003 and 8 in 2004). Three P. obsoleta males were captured in December, January, and February (one each month) (Figure 3). The single male P. menetriesi was captured the second week of May.

## Discussion

Three species of Phyllophaga were captured during the study: P. lissopyge, P. menetriesi, and P. obsoleta. This information expands the geographical distribution of P. lissopyge to the country of Colombia and continent of South America. Bates (1888) described this species from two specimens collected at Volcán Chiriqui, Panama and Chontales, Nicaragua. Morón (2001) reported the occurrence of this species from between southern Costa Rica to western Panama. In 2003, Morón (2003) expanded the range of P. lissopyge from central Nicaragua to central Panama (Figure 1). Phyllophaga lissopyge is usually found on mountain slopes (620 to 2136 m elevation) with cloud forests, tropical rain forest, and coffee plantations (Morón 2003). Phyllophaga lissopyge may have arrived in Colombia through natural or human-mediated dispersal. Phyllophaga lissopyge could have spread from Panama to the north of Chocó department through the Darien Gap, and then through the same mountain system to Antioquia. Human-mediated dispersal is also likely, the commercial interchange between Colombia and Panama in this area being very intense and unregulated. Peck et al. (2001) suggested the same route of invasion for Prosapia simulans (Walker) (Homoptera: Cercopidae) from Central America to Colombia.

Phyllophaga lissopyge is a member of the genus Phyllophaga (sensu stricto), and is one of 865 species of Phyllophaga (s. lato) recorded from the New World (Evans and Smith 2009). However, based on phylogenetic analysis of external morphological and genitalic characteristics, Coca-Abia (2002) proposed the re-establishment of the genus Trichesthes (Erichson 1847) and removed 38 species from the Phyllophaga and into Trichesthes, including P. lissopyge and P. gigantea.

The flight patterns of P. lissopyge in Central America (Morón 2001, 2003) mirror our observations in Colombia, in that adults fly from February to November, with the largest flights occurring during March, April, and May.

## References

[bibr01] Alm SR, Dawson CG, Robbins P (2004). Optimization of a valine:isoleucine methyl ester pheromone blend and comparison of Robbins and Trécé traps for the capture of Phyllophaga anxia (Coleoptera: Scarabaeidae) in Rhode Island.. Journal of Economic Entomology.

[bibr02] Bates HW., Godman FD, Salvin O (1886–1890). Pectinicornia and Lamellicornia.. Biologia Centrali Americana, 2, part 2.

[bibr03] Coca-Abia MM (2002). Reestablishment of the genus Trichesthes Erichson, 1847 (Coleoptera: Scarabaeidae, Melolonthinae) based on phylogeny.. Journal of the New York Entomological Society.

[bibr04] Espinosa-Islas A, Morón RMA, Sánchez AH, Bautista HN, Romero NJ (2005). Complejo gallina ciega (Coleoptera: Melolonthidae) asociado con céspedes en Montecillo, Texcoco, estado de México.. Folia Entomologica Mexicana.

[bibr05] Evans AV, Smith ABT (2009). An electronic checklist of the New World chafers (Coleoptera: Scarabaeidae: Melolonthinae). Version 3.. http://www.museum.unl.edu/research/entomology/SSSA/nwmelos.htm.

[bibr06] King ABS (1984). Biology and identification of white grubs (Phyllophaga) of economic importance in Central America.. Tropical Pest Management.

[bibr07] King ABS, Shannon PJ, M Carballo (1996. 
A.). Biología y identificación de (Phyllophaga) de importancia de economica en América Central.. Biología y Control de Phyllophaga spp..

[bibr08] King ABS, Shannon PJ, Carballo M (1996. 
B.). Biología, identificación y distribución de especies económicas de Phyllophaga en América Central.. Biología y Control de Phyllophaga spp..

[bibr09] Leal WS, Sawada M, Matsuyama S, Kuwahara Y, Hasegawa M (1993). Unusual periodicity of sex pheromone production in the large black chafer Holotrichia parallela.. Journal of Chemical Ecology.

[bibr10] Leal WS, Oehlschlager AC, Zarbin PHG, Hidalgo E, Shannon PJ, Murata Y, Gonzalez L, Andrade R, Ono M (2003). Sex pheromone of the scarab beetle Phyllophaga elenans and some intriguing minor components.. Journal of Chemical Ecology.

[bibr11] Londoño ME (1993). Posibilidades del control biológico en el manejo de la chisa (Coleoptera: Scarabaeoidea) para el departamento de Antioquia.. Miscelánea Sociedad Colombiana de Entomología.

[bibr12] Morón MÁ (1986). El genero Phyllophaga en México : morfologia, distribucion y sistematica supraespecifica (Insecta: Coleoptera)..

[bibr13] Morón MÁ (2001). Revision of the rugipennis group of Phyllophaga (sensu stricto) (Coleoptera: Melolonthidae).. Annals of the Entomological Society of America.

[bibr14] Morón MÁ, Melic A (2003). Las especies de Phyllophaga (s. str.) del grupo rugipennis (Coleoptera: Melolonthidae).. Escarabaeidos de Latinoamérica: Estado del conocimiento: Monografías tercer milenio.

[bibr15] Morón MÁ (2006). Revisión de las especies de Phyllophaga (Phytalus) grupos obsoleta y pallida (Coleoptera: Melolonthidae: Melolonthinae).. Folia Entomologica Mexicana.

[bibr16] Nojima S, Robbins PS, Salsbury GA, Morris BD, Roelofs WL, Villani MG (2003). L-leucine methyl ester: The female-produced sex pheromone of the scarab beetle Phyllophaga lanceolata.. Journal of Chemical Ecology.

[bibr17] Ortega-Ojeda CA, Melo-Melina EL, Gaigal A (2997). Densidad letal de Phyllophaga menetriesi (Coleoptera: Melolonthidae) sobre plantas de yuca (Manihot esculenta).. Revista Colombiana de Entomología.

[bibr18] Pardo-Locarno LC (2002). Aspectos sistemáticos y bioecológicos del complejo chisa (Col: Melolonthidae) de Caldono, Norte del Cauca, Colombia..

[bibr19] Pardo-Locarno LC, Montoya-Lerma J, Bellotti AC, Van Schoonhoven A (2005). Structure and composition of the white grub complex (Coleoptera: Scarabaeidae) in agroecological systems in Northern Cauca, Colombia.. Florida Entomologist.

[bibr20] Peck DC, Castro U, López F, Morales A, Rodríguez J (2001). First records of the sugar cane and forage grass pest, Prosapia simulans (Homoptera: Cercopidae), from South America.. Florida Entomologist.

[bibr21] Restrepo-Giraldo H, Morón MÁ, Vallejo F, Pardo-Locarno LC, Lopez-Avila A (2003). Catalogo de Coleoptera (Scarabaeidae Pleurosticti) de Colombia.. Folia Entomologica Mexicana.

[bibr22] Robbins PS, Crocker RL, Nojima S, Morris BD, Roelofs WL, Villani MG (2003). Methyl 2-(methylthio)benzoate: the unique sulfur-containing sex pheromone of Phyllophaga crinita.. Naturwissenschaften.

[bibr23] Robbins PS, Alm SR, Armstrong CD, Averill AL, Baker TC, Bauernfiend RJ, Baxendale FP, Braman SK, Brandenburg RL, Cash DB, Couch GJ, Cowles RS, Crocker RL, DeLamar ZD, Dittl TG, Fitzpatrick SM, Flanders KL, Forgatsch T, Gibb TJ, Gill BD, Gilrein DO, Gorsuch CS, Hammond AM, Hastings PD, Held DW, Heller PR, Hiskes RT, Holliman JL, Hudson WG, Klein MG, Krischik VL, Lee DJ, Linn Jr. CE, Luce NJ, MacKenzie KE, Mannion CM, Polavarapu S, Potter DA, Roelofs WL, Royals BM, Salsbury GA, Schiff NM, Shetlar DJ, Skinner M, Sparks BL, Sutschek JA, Sutschek TP, Swier SR, Sylvia MM, Vickers NJ, Vitium PJ, Weidman RB, Weber DC, Williamson RC, Villani MG (2006). Trapping Phyllophaga spp. (Coleoptera: Scarabaeidae: Melolonthinae) with sex attractants in the United States and Canada.. Journal of Insect Science.

[bibr24] Robbins PS, Cash DB, Linn CE, Roelofs WL (2008). Experimental evidence for three pheromone races of the scarab beetle Phyllophaga anxia (LeConte).. Journal of Chemical Ecology.

[bibr25] Robbins PS, Nojima S, Polavarapu S, Koppenhöfer A, Rodriguez-Saona C, Holdcraft RJ, Consolie NL, Peck DC, Roelofs WL (2009). Sex pheromone of the scarab beetle Phyllophaga (Phytalus) georgiana (Horn).. Journal of Chemical Ecology.

[bibr26] Salvadori JR, Oliviera LJ (2001). Manejo de corós em lavouras sob plantio direto..

[bibr27] Salvadori JR, Silva MT, Salvadori JR, Avila CJ, Silva MT (2004). Coró-dotrigo..

[bibr28] Serna-Patino LM (2004). Reconocimiento de especies del complejo chisa (Coleoptera-Melolonthidae) asociados a los cultivos de yuca y pasto en el municipio de Pereira y Alrededores..

[bibr29] Vallejo F, Morón MÁ, Orduz S (1998). First report and description of immature stages of Phyllophaga obsoleta (Blanchard) (Coleoptera: Melolonthidae) in Colombia.. Coleopterists Bulletin.

[bibr30] Villegas NP, Gaigl A, Vallejo LF (2006). Reconocimiento de especies del complejo chisa (Coleoptera: Melolonthidae) ascoiadas al cultivo de cebolla y pasto kikuyo del departameno de Risaralda, Colombia.. Agronomia.

[bibr31] Villegas NP, Gaigal A, Vallejo LF (2008). The white grub complex (Coleoptera: Melolonthidae) associated with onion and pasture in Risaralda, Colombia.. Revista Colombiana de Entomología.

[bibr32] Zarbin PHG, Leal WS, Ávila CJ, Oliveira LJ (2007). Identification of the sex pheromone of Phyllophaga cuyabana (Coleoptera: Melolonthidae).. Tetrahedron Letters.

[bibr33] Zhang A, Robbins PS, Leal WS, Linn CE, Villani MG, Roelofs WL (1997). Essential amino acid methyl esters: major sex pheromone components of the cranberry white grub, Phyllophaga anxia (Coleoptera: Scarabaeidae).. Journal of Chemical Ecology.

